# Endophytic *Beauveria bassiana* promotes plant biomass growth and suppresses pathogen damage by directional recruitment

**DOI:** 10.3389/fmicb.2023.1227269

**Published:** 2023-08-16

**Authors:** Li Sui, Yang Lu, Linyan Zhou, Nannan Li, Qiyun Li, Zhengkun Zhang

**Affiliations:** ^1^Institute of Plant Protection, Jilin Academy of Agricultural Sciences, Gongzhuling, Jilin, China; ^2^College of Plant Protection, Jilin Agricultural University, Changchun, China; ^3^Jilin Agricultural Science and Technology University, Jilin, China

**Keywords:** entomopathogenic fungi, endophytic, promote plant growth, phytopathogen, recruitment function

## Abstract

**Introduction:**

Entomopathogenic fungi (EPF) can colonize and establish symbiotic relationships with plants as endophytes. Recently, EPF have been reported to suppress plant pathogens and induce plant resistance to diseases. However, the potential mechanisms via which EPF as endophytes control major plant diseases in situ remain largely unknown.

**Methods:**

Pot and field experiments were conducted to investigate the mechanisms *via* which an EPF, *Beauveria bassiana*, colonizes tomato, under *Botrytis cinerea* infection stress. *B. bassiana* blastospores were inoculated into tomato plants by root irrigation. Tomato resistance to tomato gray mold caused by *B. cinerea* was evaluated by artificial inoculation, and *B. bassiana* colonization in plants and rhizosphere soil under *B. cinerea* infection stress was evaluated by colony counting and quantitative PCR. Furthermore, the expression levels of three disease resistance-related genes (*OXO*, *CHI*, and *atpA*) in tomato leaves were determined to explore the effect of *B. bassiana* colonization on plant disease resistance performance in pot experiments.

**Results:**

*B. bassiana* colonization could improve resistance of tomato plants to gray mold caused by *B. cinerea*. The incidence rate, lesion diameter, and disease index of gray mold decreased in both the pot and field experiments following *B. bassiana* colonization. *B. bassiana* was more likely to accumulate in the pathogen infected leaves, while decreasing in the rhizosphere soil, and induced the expression of plant resistance genes, which were up-regulated in leaves.

**Discussion:**

The results indicated that plants could “recruit” *B. bassiana* from rhizosphere soil to diseased plants as directional effects, which then enhanced plant growth and resistance against pathogens, consequently inhibiting pathogen infection and multiplication in plants. Our findings provide novel insights that enhance our understanding of the roles of EPF during pathogen challenge.

## 1. Introduction

Entomopathogenic fungi (EPF) have recently been demonstrated to have a capacity to colonize a wide array of plant species as endophytes, and their potential capacity to control plant pathogens and insect pests (Jaber and Ownley, 2017; Sinno et al., 2021; Altaf et al., 2023) as well as to improve plant growth has increasingly attracted the attention of researchers in recent years (Castillo López and Sword, 2015; Jaber and Enkerli, 2016; Sui et al., 2020). *Beauveria bassiana* Vuillemin (Ascomycota: Hypocreales), an endophyte (fungus or bacterium occurring inside plant tissues without causing any apparent symptoms, Wilson, 1995), is the most extensively studied entomopathogen. Endophytic *B. bassiana* reportedly suppresses *Rhizoctonia solani* and *Pythium myriotylum* growth in tomato (Ownley et al., 2004) and cotton (Ownley et al., 2008), Zucchini yellow mosaic virus colonization in squash (Jaber and Salem, 2014), and *Plasmopara viticola* colonization in grapevines (Jaber, 2015). Despite the substantial evidence available on the antagonistic activity of endophytic EPF against phytopathogens (Ownley et al., 2010; Sui et al., 2022), such activity remains understudied when compared with their recognized biocontrol activity against insect pests. Investigation of the biocontrol activity of *B. bassiana* against phytopathogens, and elucidating its mode of action against disease-causing organisms, could enhance the biopesticide potential of the fungus greatly.

*B. bassiana* can colonize different plants through various inoculation methods, including seed dressing, root irrigation, stem injection, and foliar spray (Vega, 2008). For example, Jaber and Enkerli (2016) demonstrated that *B. bassiana* could systemically colonize different plant parts and improve plant growth when applied as a seed dressing agent. Similar results were reported in wheat that was inoculated using the seed dressing and soil treatment methods; as an endophytic microbe, *B. bassiana* was able to spread from the inoculation sites to plant tissues and survive in the tissues (Sánchez-Rodríguez et al., 2018). Previous studies have used different methods to evaluate endophyte activity in plant leaves, stems, and roots (Tefera and Vidal, 2009; Parsa et al., 2013). Wagner and Lewis (2000) used light and electron microscopy to observe the penetration of mycelium formed by germinated conidia of *B. bassiana* through maize leaves, and the process by which they entered and grew in the plants. Other researchers have confirmed *B. bassiana* colonization in plants using scanning electron microscopy and molecular biology techniques, providing a convenient method for further determination of its endogenous activity (Landa et al., 2013; Behie et al., 2015). Recently, the distribution characteristics of *B. bassiana* in maize plant tissues have been clarified using green fluorescent protein (GFP)-labeled strains, which has provided technical support for further exploration of endophytic bacteria colonization in plant tissues (Sui et al., 2022). In summary, numerous studies have demonstrated that *B. bassiana* in plant tissues is symbiotic; however, a clear understanding of the preferential localization within plant tissues is still lacking.

Plants frequently harbor fungi asymptomatically within their tissues, and endophytic *B. bassiana* forms associations with multiple plant species both below and above ground (McKinnon et al., 2017). In recent years, several articles have focused on the biological processes involved in plant host colonization, and establishment and persistence of the endophytic stage (Wagner and Lewis, 2000; Landa et al., 2013; Quesada-Moraga, 2020). Endophytic fungi display preferential tissue colonization within their plant hosts and benefit plant growth. According to Tefera and Vidal (2009), inoculation method and plant growth medium influence endophytic colonization. In addition, according to Behie et al. (2015), *Metarhizium* is restricted to plant roots, whereas *B. bassiana* is observed throughout a plant. Furthermore, McKinnon et al. (2018) demonstrated plant defense responses under wounding stress, resulting in the apparent recruitment of *Beauveria* in the rhizosphere, which might be an indirect defensive strategy against stress and/or the result of induced systemic susceptibility in microbe colonization. Moreover, numerous studies have uncovered plant resistance genes related to nutrient uptake and biotic and abiotic stress resistance, which may influence the composition and function of microbial communities, while suggesting the existence of robust recognition and defense mechanisms (Gururani et al., 2012; Liu et al., 2022). Although various studies have reported that *B. bassiana* can colonize a broad range of plant hosts as an endophyte, few studies have considered the factors regulating EPF colonization under different conditions, especially biotic stress.

Understanding the endophytic functions of *B. bassiana* is crucial for determination of how the fungus influences host plant responses to ecological factors or any stress. *Botrytis cinerea* is a common and major phytopathogen that causes gray mold or blight disease in over 200 plant species, including many economically important fruits, vegetables, and other food crops. The pathogen often infects leaves, stems, flowers, and fruits of the host plants (Williamson et al., 2007). Sui et al. (2022) observed that compared with aerial conidia, hydrophilic blastopores of *B. bassiana* more effectively defend against *B. cinerea*. Although the positive effects of EPF in plants have been documented extensively (Vega, 2018; Quesada-Moraga, 2020), whether and how EPF influence plant resistance to biotic stress remains largely unknown. Therefore, the aim of the present study was to investigate potential interactions between EPF and plants under phytopathogen stress, we explore the regulation of the distribution of *B. bassiana* in tomato tissue under *B. cinerea* stress. The authors hypothesize that the endophytic *B. bassiana* can be regulated in plant tissue and has bottom-up and directional effects on plant growth and resistance.

## 2. Materials and methods

### 2.1. Fungal strain and conidial suspension preparation

A transformant of *B. bassiana* (BbOFDH1-5-GFP) that expresses green fluorescent protein was used in the present study. The strain was deposited in the China General Microbiological Culture Collection, with accession number CGMCC. 15673. BbOFDH1-5-GFP was integrated with plasmid pABeG containing phosphinothricin resistance gene (*bar*) and enhanced green fluorescence protein gene (*egfp*), using the wild-type *B. bassiana* strain BbOFDH1-5 (GenBank No. ANFO01) and the blastospore transformation method. Blastospores used in the present study were produced in a Sabouraud dextrose medium with yeast extract (SDY) liquid culture, for 120 h at 26°C and 160 rpm (Sui et al., 2022). Afterward, the harvested conidia were filtered using a sterile syringe and cotton wool to remove hyphal debris and obtain a clean stock suspension, and then the suspension was suspended in sterile water by adjusting the initial stock concentration to a final concentration of 1 × 10^8^ conidia mL^−1^.

### 2.2. Plant material origin and preparation

Tomato (*Solanum lycopersicum* var. BEAUTY) seeds were obtained from Jilin Mainland Seed Industry Co. LTD, Gongzhuling, Jilin, China. Seeds were washed in 1% sodium hypochlorite for 3 min, followed by 2 min in 75% ethanol, after which they were rinsed thrice in sterile water (Sui et al., 2020). Following surface sterilization, seeds were sown in 10 cm × 8 cm seedling pots filled with sterilized field soil (Supplementary Figure S1a). Prior to planting, the soil was autoclaved twice for 2 h (leaving one day between autoclaving), and aerated and mixed to avoid trapping gasses toxic to microbiota and plants (Trevors, 1996). Seeds were sown in the greenhouse directly for use in the field experiments. Seedlings were watered at 5–6 days intervals in both experiments with sterilized water.

### 2.3. Plant inoculation with pathogen

*B. cinerea* was provided by Prof. Wei Li, College of Plant Protection, Hunan Agricultural University, Hunan, China. *B. cinerea* was cultured on potato dextrose agar (PDA) medium at 26°C for a week before inoculation. Fungus agar blocks were obtained with a sterile hole puncher (0.5 cm in diameter) for use in inoculation. After 48 h of inoculation with *B. bassiana*, each of the 3rd entire fully developed leaflets of tomato plants in *B. cinerea* treatments were inoculated with a *B. cinerea* block at 23°C and 95% relative humidity (RH), and then covered with plastic wrap for moisturization (Sun et al., 2019).

### 2.4. Plant inoculation with *Beauveria bassiana* and endophytic colonization

Both the pot and field experiments were set up based on a completely randomized design comprising four treatments, with four replicates, including control, tomato inoculation with *B. cinerea* (Bc); tomato inoculation with *B. bassiana* suspension (BS); and tomato inoculation with both *B. bassiana* suspension and *B. cinerea* (BS + Bc). The pot experiments had 20 pots per replicate for each of the four treatments, whereas the field experiments had 15 plants per replicate for each of the four treatments. To establish *B. bassiana* as an endophyte in tomato, two treatments were combined, including seed immersion and soil drench inoculation. Tomato seeds were immersed in a *B. bassiana* blastospore suspension (1 × 10^8^ conidia ml^−1^ in sterile water) for 12 h. For soil drench inoculations, 20 mL of *B. bassiana* blastospore suspension (1 × 10^8^ conidia ml^−1^ in sterile water) was applied at the four leaves stage. For the two treatments not inoculated with *B. bassiana*, the seeds were immersed in sterile water, or the soil was drenched with the same amount of sterile water.

*B. bassiana* colonization of tomato leaves were assessed by plating surface-sterilized leaf segments on PDA 24 h post soil drench inoculations, which is described in detail in Sui et al. (2020). Nine 1 cm × 1 cm segments of leaves were removed from each plant in a total of 10 plants per treatment in the pot experiments. Plant colonization by endophytic *B. bassiana* was considered to have occurred if one or more leaf pieces per plant individual produced *B. bassiana* outgrowth. Colonization rates were calculated as follows: colonization rate = 100% × (the number of *B. bassiana* colonized plants/total number of plants).

### 2.5. Effects of endophytes on plant growth

To evaluate the effects of inoculated EPF on tomato growth, root length and seeding height were assessed in pot experiments, while fruit number and fruit weight per plant were assessed in field experiments. Any soil adhering to the roots obtained after 3–5 days of seedling emergence were thoroughly removed, and the root lengths of tomato seedings were measured from the plant base to the root tip. Tomato plant height was measured from the soil surface to the shoot tip at 7, 14, and 21 days post seedling emergence. When the tomatoes entered the picking period in the field experiment, they were picked every 3 days and weighed to calculate the yield, until the end of harvest; the number of fruits and fruit weight per plant were also determined (Bogiani et al., 2008).

### 2.6. Gray mold incidence assessment

The resistance of plants to *B. cinerea* was assayed based on the incidence rate, diameter of ensuing lesion, and disease index in both the pot and field experiments. At 6 days post inoculation (dpi), the disease incidence in the tomato plants was calculated as follows: Incidence rate (%) = plants with disfigured spots/total number of tomato plants × 100% (Li T. et al., 2022). The diameter of ensuing lesion(s) was investigated at 6 dpi. The horizontal and vertical diameters of ensuing lesions, which represented the diameters of the plaques, were measured and their mean values calculated. Gray mold was evaluated on a scale of 0–4 with: 0 = no necrosis, leaf area is completely healthy; 1 = 25% of the leaf area exhibited symptoms; 2 = 50% of the leaf area exhibited symptoms; 3 = 75% of the leaf area exhibited symptoms; 4 = 100% of leaf area exhibited *Botrytis* symptoms. A disease index was calculated as the areas of the inoculated leaves, and each treatment was applied to 10 plants (Ben-Shalom et al., 2003).

### 2.7. Isolation of *Beauveria bassiana* from soil specimens

Tomato root rhizosphere soil was sampled at 3 days post *B. cinerea* inoculation in pot experiments. Plants were first carefully extracted from their pots and shaken gently to remove excess and/or loose soil. The roots were then brushed gently with a sterile paintbrush to remove the rhizosphere soil onto sterilized trays. Rhizosphere soil was then mixed aseptically by hand within the trays (McKinnon et al., 2018). Five plants of root adhered soil per replicate were selected randomly and examined, for a total of 20 plants in four treatments, and 1 g sample of rhizosphere soil was taken from each plant for the detection of *B. bassiana* colonies in soil. The plate colony counting method (Mitsuaki and Hiroki, 1996) was used to determine *B. bassiana* colony number. Soil specimens were suspended in 0.05% Tween 80 in a 1 g/200 mL concentration, and suspensions were spread using a glass rod on PDA plate at a 0.2 mL/plate concentration. Plates were incubated at 25°C in total darkness and the numbers of colonies on the media were counted 5 days later. *B. bassiana* colonies obtained from PDA plates were observed under optical microscopy for morphological identification of the fungal species.

### 2.8. Quantification of *Beauveria bassiana*, pathogen, and disease resistance genes in tomato leaves by quantitative PCR

Quantitative PCR (qPCR) assays (Gachon and Saindrenan, 2004) were used to evaluate the effect of inoculated EPF on the relative expression of fungi (*B. bassiana*), pathogen (*B. cinerea*), and target resistance genes (Oxalate oxidase [*OXO*], chitinase [*CHI*], and ATP synthase [*atpA*]) in tomato leaves in the pot experiments. The leaves of tomato in different treatments were collected two times for *OXO*, *CHI* and *atpA* detection, at 5 days post *B. bassiana* soil drench inoculation and 3 days post *B. cinerea* inoculation. Five 1 cm × 1 cm segments of plant leaves were randomly selected for each treatment and stored at −80°C until processing. The relative amounts of *B. bassiana* and *B. cinerea* were determined using qPCR employing specific primers after 3 days post *B. cinerea* inoculation. Total DNA in each treatment was extracted, and the specific primers employed in qPCR are listed in Supplementary Table S1.

### 2.9. Statistical analyses

Before analysis, data were subjected to normality and homogeneity tests of variance using *qqplot*, and the Shapiro–Wilk Normality test (at 0.05 significance level). All data were subjected to one-way Analysis of Variance, the Tukey’s test was used to make multiple comparisons of the mean (*p* < 0.05), and a paired trial was performed using the *t* test. IBM SPSS Statistics 22 (IBM Corp., Armonk, NY, United States) was used for data analysis. Figures were illustrated using SigmaPlot 12.5 (Systat Software, San Jose, CA, United States).

## 3. Results

### 3.1. Assessment of endophytic colonization in tomato seedlings

**Figure 1:**
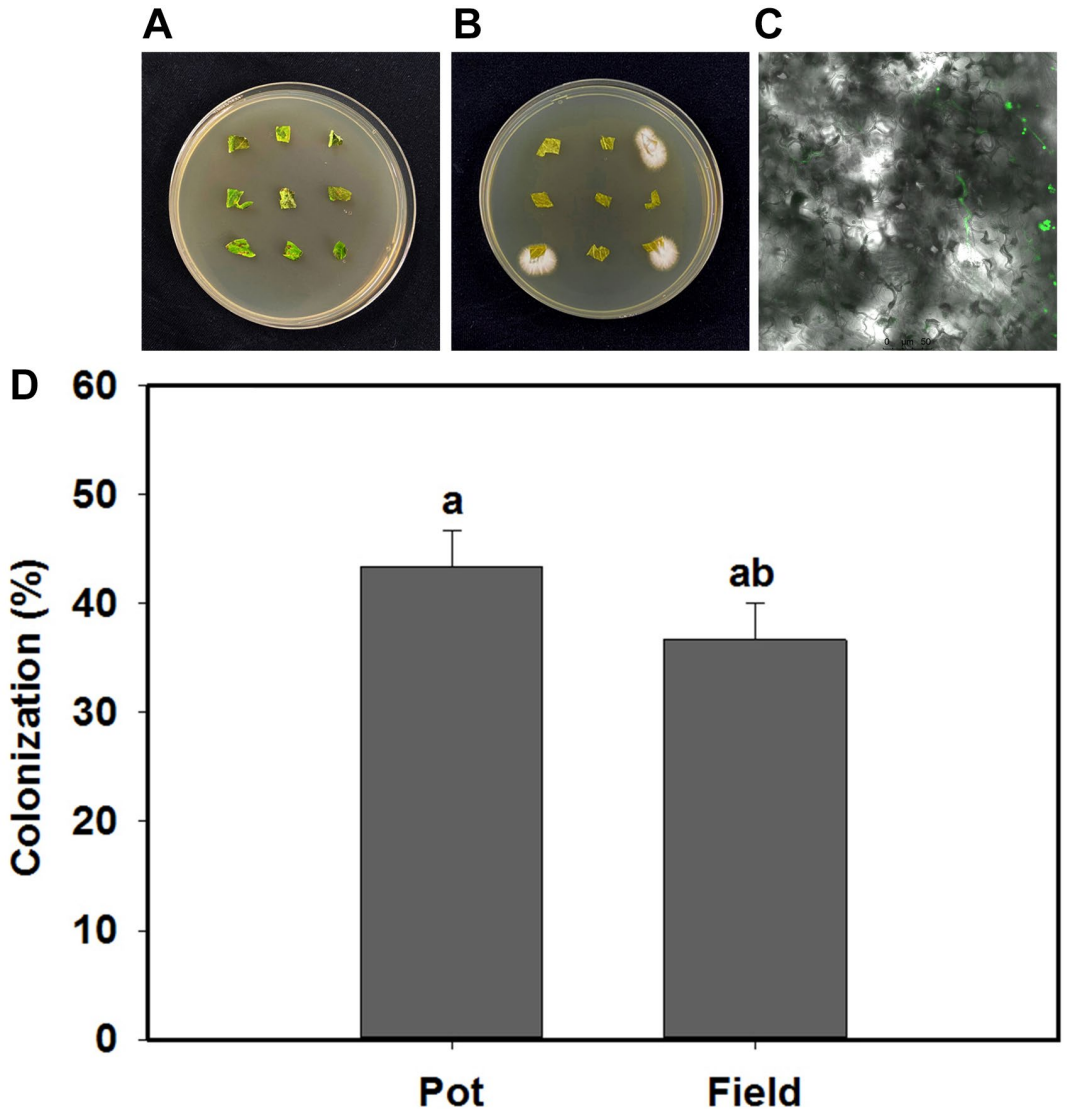
*Beauveria bassiana* colonization in tomato leaves. **(A)** Control plates without *B. bassiana* growth, **(B)** *B. bassiana* growing from three plant sections, **(C)** morphological characteristics of *B. bassiana* blastospores (BS) in tomato leaves (100×), **(D)** Colonization rates of *B. bassiana* in tomato plants at pot and field experiments. Values are means ± standard error (SE). Different letters above bars indicate significant differences between two treatments (*p* < 0.05).

*B. bassiana* endophytically colonized tomato plants in response to the inoculation treatments in the present study (Figures 1A,B). Blastospores and their germinated hyphae were observed in the leaves of tomato under light and confocal microscopy (Figure 1C). The colonization rates of *B. bassiana* in inoculated plants was 43.3 and 33.3%, respectively, in the pot and field experiments, and there was no *B. bassiana* growth in the control treatment (Figure 1D).

### 3.2. Effect of endophytic colonization on tomato growth

**Figure 2:**
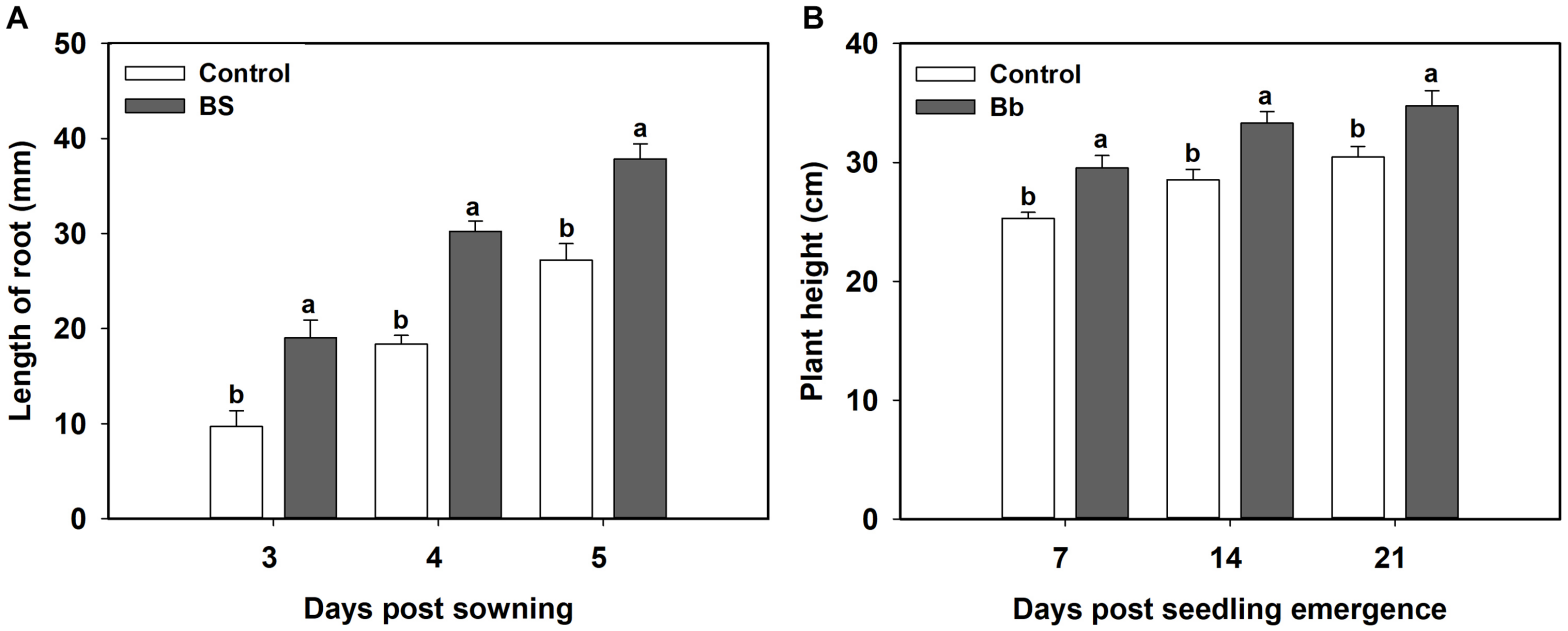
Effects of *Beauveria bassiana* (Bb) colonization on tomato seeding growth. **(A)** Root length at 3–5 days post sowing, and **(B)** plant height at 7, 14, and 21 days post seedling emergence. Values are means ± standard error (SE). Different letters above the bars indicate significant differences between the two treatments (*p* < 0.05).

*B. bassiana* had significant effects on tomato seedling root length and plant height in the pot experiments (Table 1). Root length under the BS treatment was significantly greater than those in the control treatment during 3–5 days post sowing. Mean root lengths under the BS treatments were significantly higher at 3 (*F* = 9.378, *p* = 0.014), 4 (*F* = 26.172, *p* = 0.01), and 5 (*F* = 9.152, *p* = 0.015) days post-sowing than those in the control treatment (Figure 2A). Plant heights under the BS treatment were significantly higher at 7 (*F* = 7.235, *p* = 0.025), 14 (*F* = 6.8, *p* = 0.029) and 21 (*F* = 6.476, *p* = 0.049) days post seeding emergence than those in the control treatment (Figure 2B).

**Table 1:** One-way analysis of variance of the effects of *Beauveria bassiana* (Bb) on tomato growth.

Response variables		*B. bassiana* (Bb)	
		*F*	*p*
Root length	3 d	9.378	0.014
	4 d	26.172	0.001
	5 d	9.152	0.015
Plant height	7 d	7.235	0.025
	14 d	6.8	0.029
	21 d	3.476	0.049
Quantity of fruit	/	4.156	0.048
Weight of fruit	/	4.348	0.043

**Figure 3:**
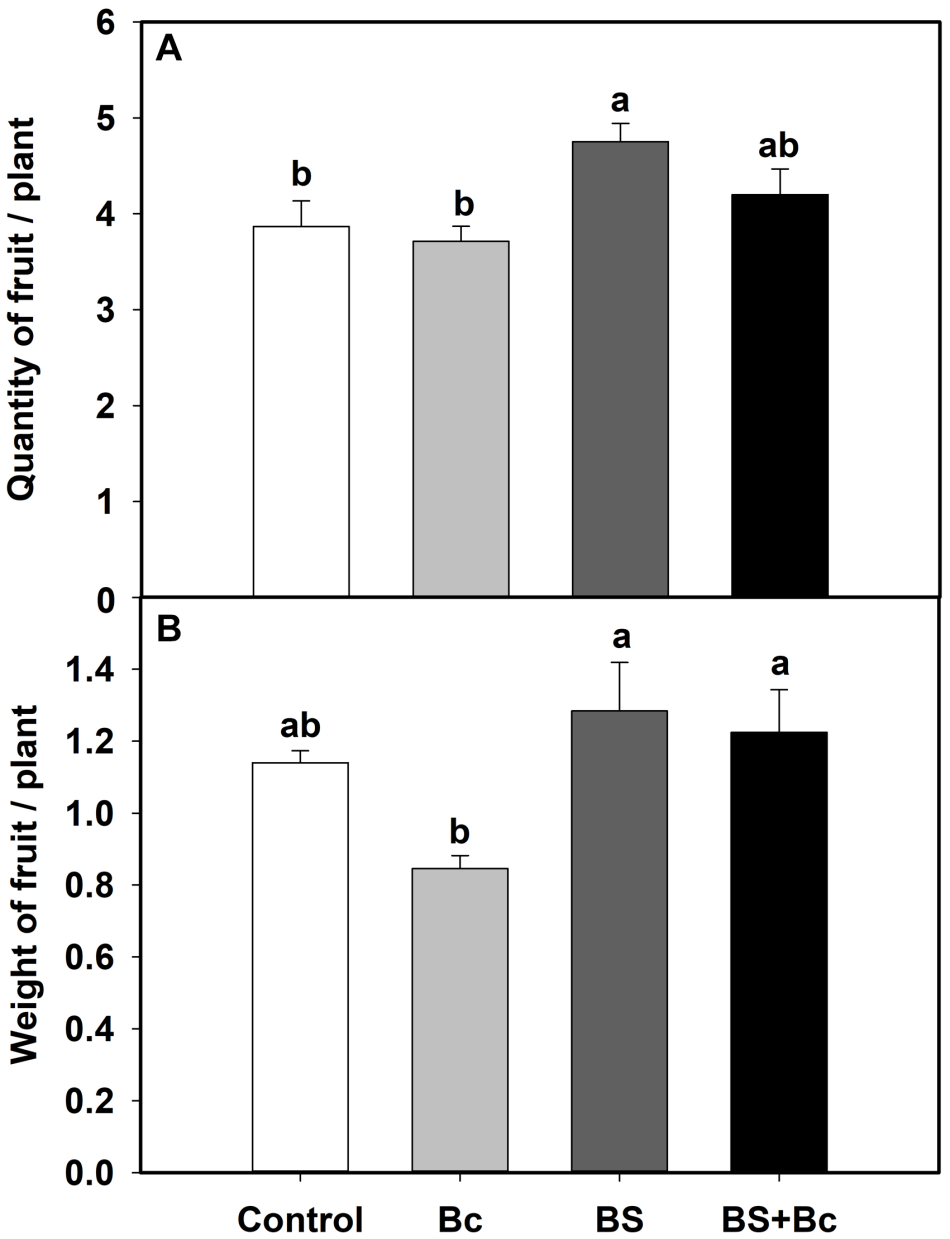
Effects of *B. bassiana* (Bb) colonization on tomato yield characteristics. **(A)** Quantity of fruits per plant, and **(B)** weight of fruits per plant. Values are means ± SE. Different letters above the bars indicate significant differences among the four treatments (*p* < 0.05).

We also observed significant effects of *B. bassiana* on tomato yield characteristics in the field experiment (Table 1). Fruit quantities in the BS treatment were significantly higher (*F* = 4.156, *p* = 0.048), by 22.9 and 28.0%, respectively, than those in the control and Bc treatments, and fruit quantity in the BS + Bc treatment was higher than that in the Bc treatment by 13.1% (Figure 3A). Fruit weights in the BS and BS + Bc treatments were significantly higher than that in the Bc treatment (*F* = 4.348, *p* = 0.043), by 12.7 and 7.4%, respectively (Figure 3B).

### 3.3. *Beauveria Bassiana* induced resistance against *Botrytis cinerea* in tomato

**Figure 4:**
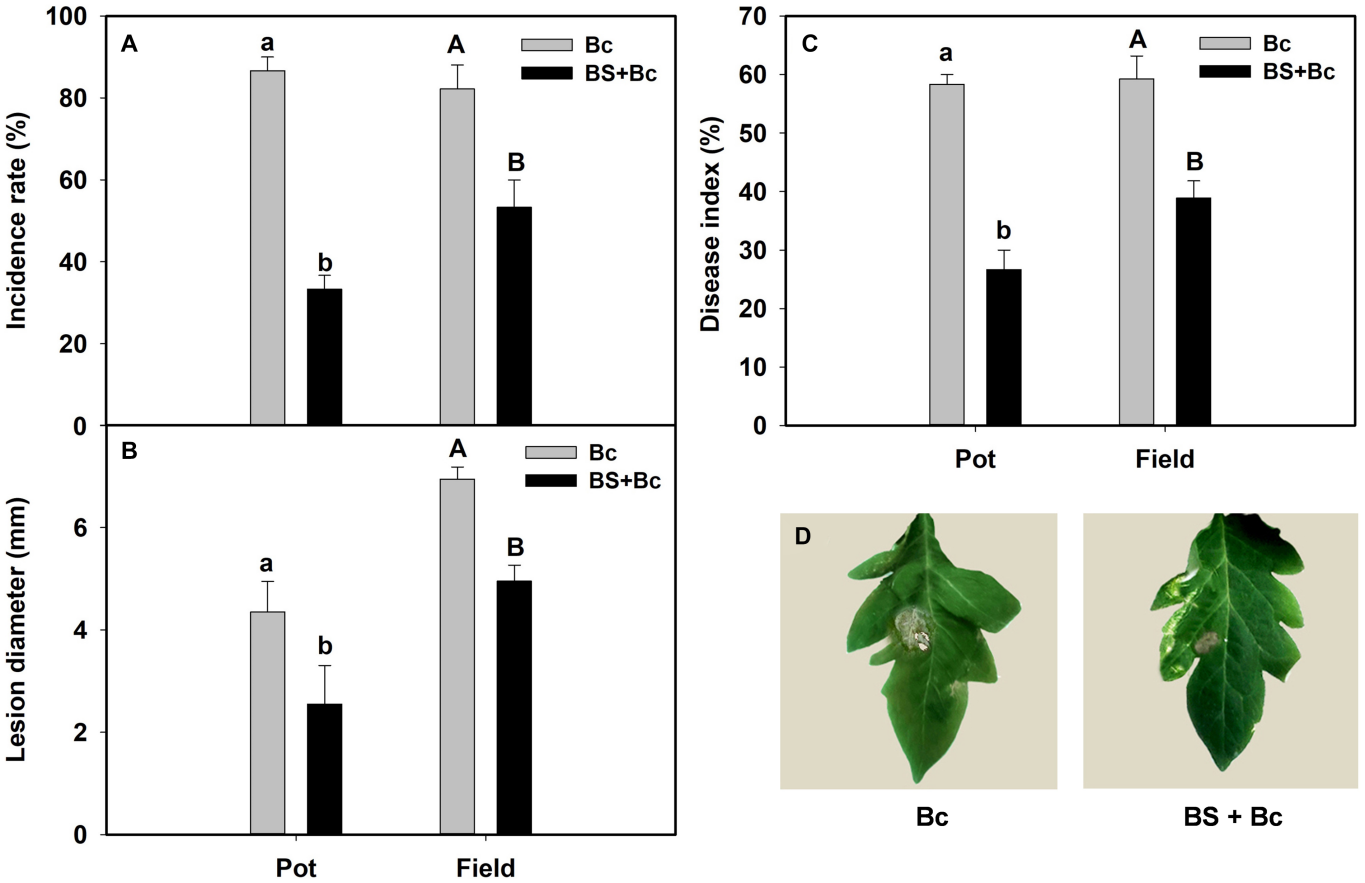
*Beauveria bassiana* colonization induced resistance against *Botrytis Cinerea* in tomato in pot and field experiments. **(A)** Incidence rate at 6 days post inoculation (dpi), **(B)** lesion diameter at 6 dpi, **(C)** disease index in different experiments, and **(D)** visual assessment of disease symptoms in different treatments at 6 dpi. Bc: tomato plants inoculated with *B. cinerea*, BS + Bc: tomato plants inoculated with blastospores of both *B. bassiana* and *B. cinerea*. Values are means ± standard error (SE). Different letters above the bars indicate significant differences between the Bc and BS + Bc treatments (*p* < 0.05).

The incidence rate, lesion diameter, and disease index in *B. bassiana*-inoculated plants were significantly lower than those in non-*B. bassiana*-treated plants in both the pot and field experiments under *B. cinerea* infection (Table 2). The Bc treatment that was inoculated with *B. cinerea* had the highest incidence rate in both the pot and field experiments, whereas the incidence rate in the BS + Bc treatment was significantly lower than that in the Bc treatment in both the pot (*F* = 258, *p* < 0.0001) and field (*F* = 66.052, *p* < 0.0001) experiments (Figure 4A). Disease symptoms were observed on tomato leaves and there were significantly smaller lesions on tomato leaves colonized by *B. bassiana* blastospores than in the Bc treatment in both the pot (*F* = 15.442, *p* = 0.004) and field (*F* = 113.216, *p* < 0.0001) experiments. Plant leaves inoculated with only *B. cinerea* had lesions of about 2.8–4.4 mm in diameter, while they were 2.0–2.5 mm on leaves colonized by *B. bassiana* at 2–4 dpi (Figures 4B,D). A significant difference in disease index was observed in both the pot (*F* = 184.2, *p* < 0.0001) and field (*F* = 251.618, *p* < 0.0001) experiments between the Bc and BS + Bc treatments, and the disease index in the BC and BS + Bc treatments were lower than that in the Bc treatment (Figure 4C).

**Table 2:** One-way analysis of variance of the effects of *Beauveria bassiana* (Bb) induced resistance in tomato against *Botrytis cinerea*.

Response variables		*B. bassiana* (Bb)	
		*F*	*p*
Infection rate	Pot	258	< 0.0001
	Field	66.052	< 0.0001
Lesion diameter	Pot	15.442	0.004
	Field	113.216	< 0.0001
Disease index	Pot Field	184.2251.618	< 0.0001 < 0.0001

### 3.4. Distribution of *Beauveria bassiana* and *Botrytis cinerea* in tomato plants

**Figure 5:**
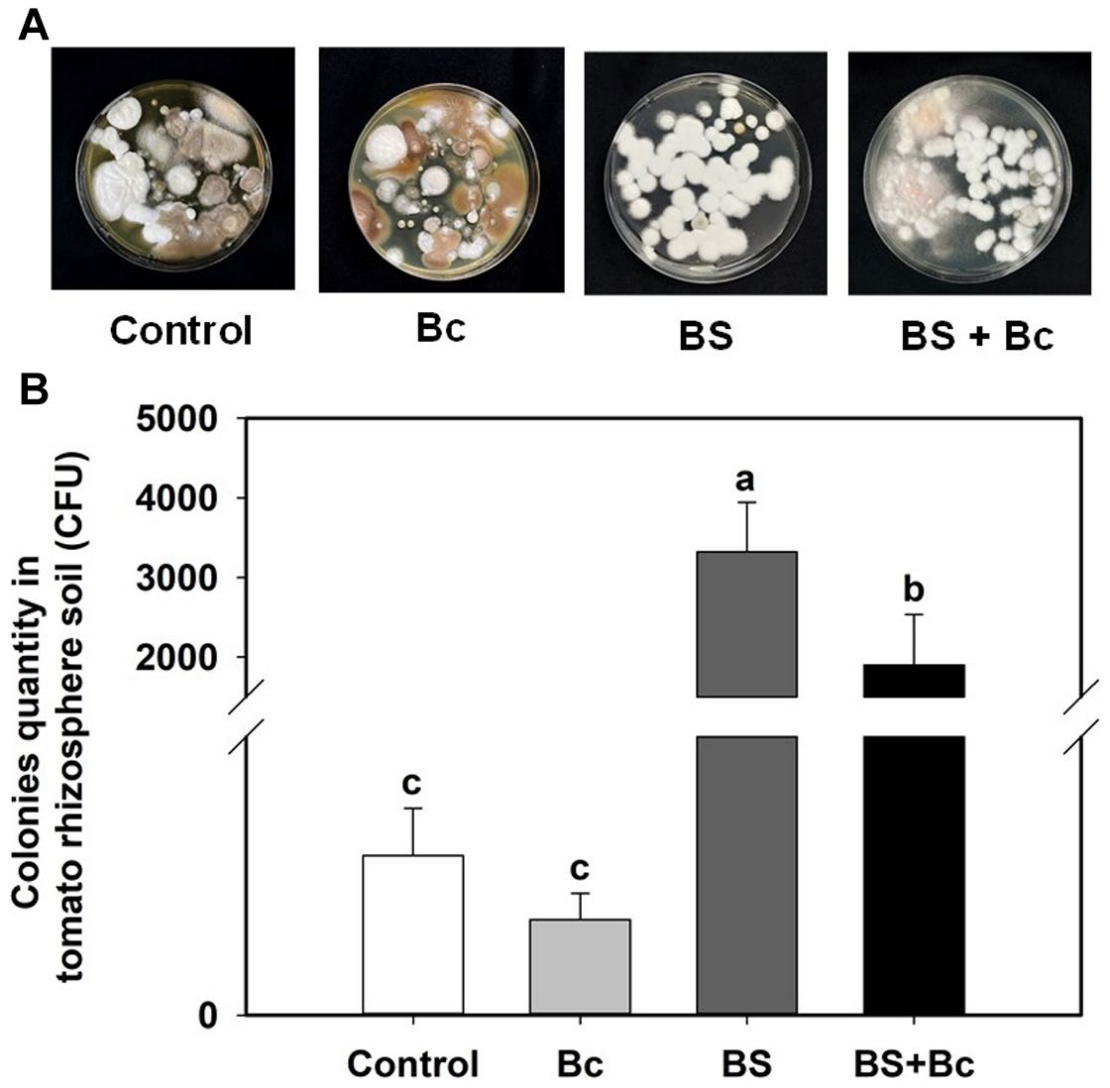
*Beauveria bassiana* colonies in tomato rhizosphere soil at 3 days post *Botrytis cinerea* inoculation. **(A)** *B. bassiana* colonies in tomato rhizosphere soil by Potato Dextrose Agar detection, and **(B)** *B. bassiana* colonies in tomato rhizosphere soil. Values are means ± standard error (SE). Different letters above the bars indicate significant differences between the two treatments (*p* < 0.05).

The *B. bassiana* colonies cultured on PDA plates from the rhizosphere soil of tomato at 3 dpi in the Bc, BS, and BS + Bc treatments were significantly different (*F* = 9.085, *p* = 0.006) (Figure 5A). *B. bassiana* colony quantity in the BS + Bc treatment was significantly lower than that in the BS treatment by 42.7% (Figure 5B).

**Figure 6:**
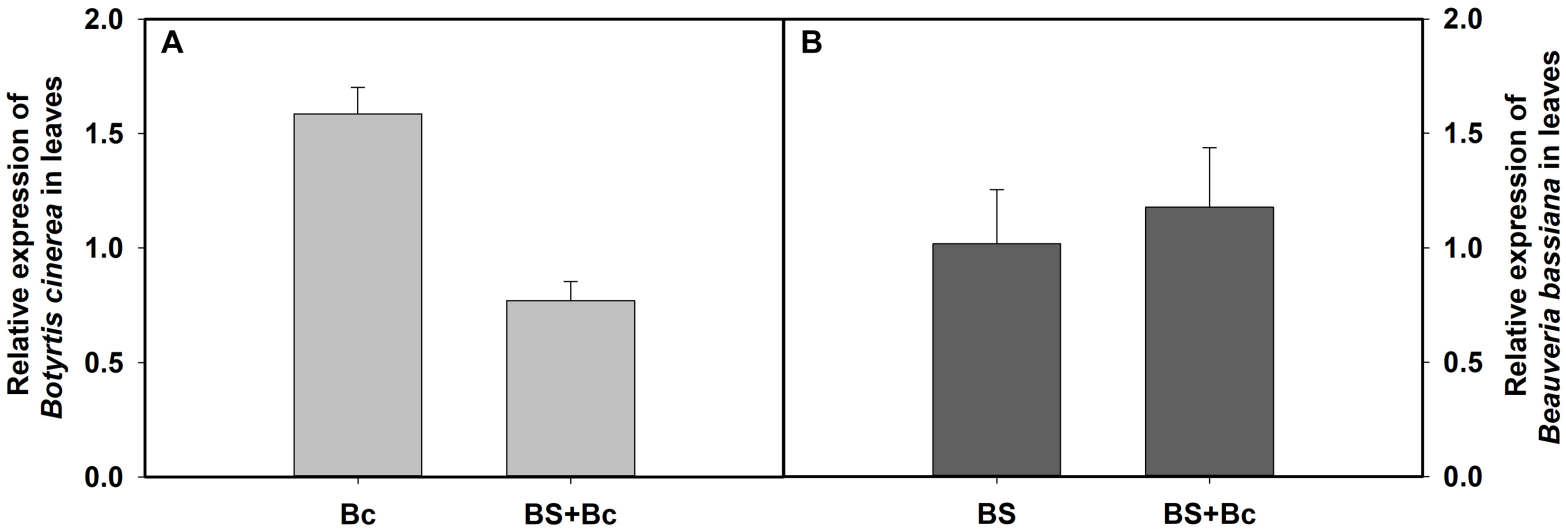
Relative expression level of *Botrytis cinerea* and *Beauveria bassiana* in tomato leaves at 3 days post *B. cinerea* inoculation. **(A)** Relative expression level of *B. cinerea* in tomato leaves, and **(B)** relative expression level of *B. bassiana* in tomato leaves.

Our results showed that the relative expression level of *B. cinerea* in the Bc treatment was 2.06 times compared with that in the BS + Bc treatment at 3 days post *B. cinerea* inoculation (Figure 6A), whereas the relative expression level of *B. bassiana* in the BS treatment was lower than that in the BS + Bc treatment (Figure 6B).

### 3.5. Quantification of disease resistance genes in tomato leaves

**Figure 7:**
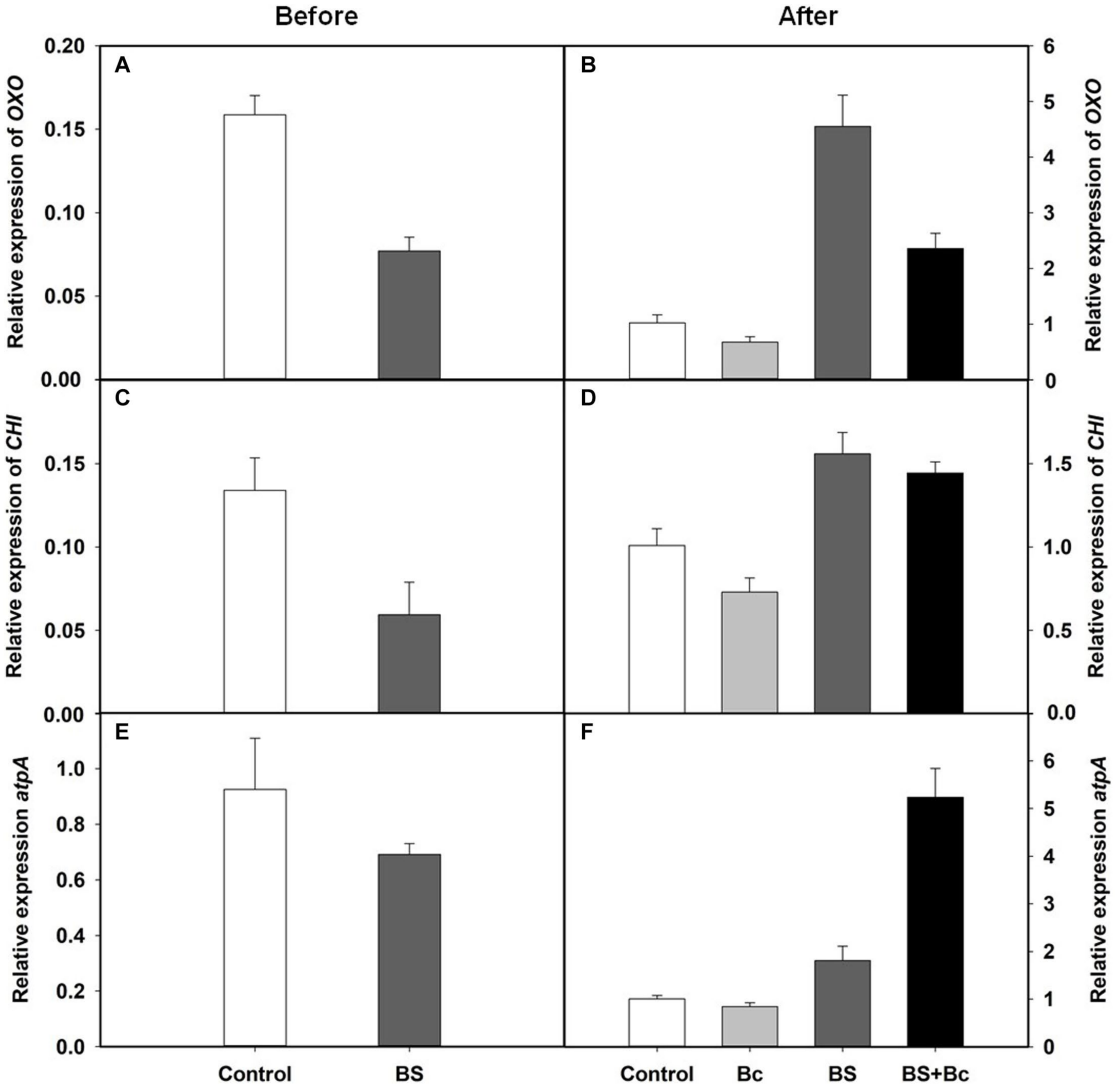
Relative expression levels of disease resistance related genes in tomato leaves. **(A)** Relative expression levels of Oxalate oxidase (*OXO*) gene in tomato leaves before *Botrytis cinerea* inoculation, **(B)** relative expression levels of *OXO* in tomato leaves at 3 days post *B. cinerea* inoculation, **(C)** relative expression levels of chitinase (*CHI*) gene in tomato leaves before *B. cinerea* inoculation, **(D)** relative expression levels of *CHI* in tomato leaves at 3 days post *B. cinerea* inoculation, **(E)** relative expression levels of ATP synthase (*atpA*) gene in tomato leaves before *B. cinerea* inoculation, and **(F)** relative expression levels of *atpA* in tomato leaves at 3 days post *B. cinerea* inoculation.

We observed that the relative expression levels of disease resistance related genes, Oxalate oxidase (*OXO*), chitinase (*CHI*), and ATP synthase (*atpA*), in the BS treatment were lower than those in the control treatment before *B. cinerea* inoculation (Figures 7A,C,E), and were 3.46, 1.54, and 1.80 times higher, respectively, in the BS treatment, when compared with those in the control treatment 3 days post *B. cinerea* inoculation (Figures 7B,D,F). In addition, the relative expression levels of the three disease resistance genes, *OXO*, *CHI*, and *atpA*, were extremely higher, by 3.51, 1.98, and 6.2 times, respectively, in the BS + Bc treatment, when compared with those in the Bc treatment, while they were lower in the Bc treatment than in the control treatment 3 days post *B. cinerea* inoculation (Figures 7B,D,F).

## 4. Discussion

**Figure 8:**
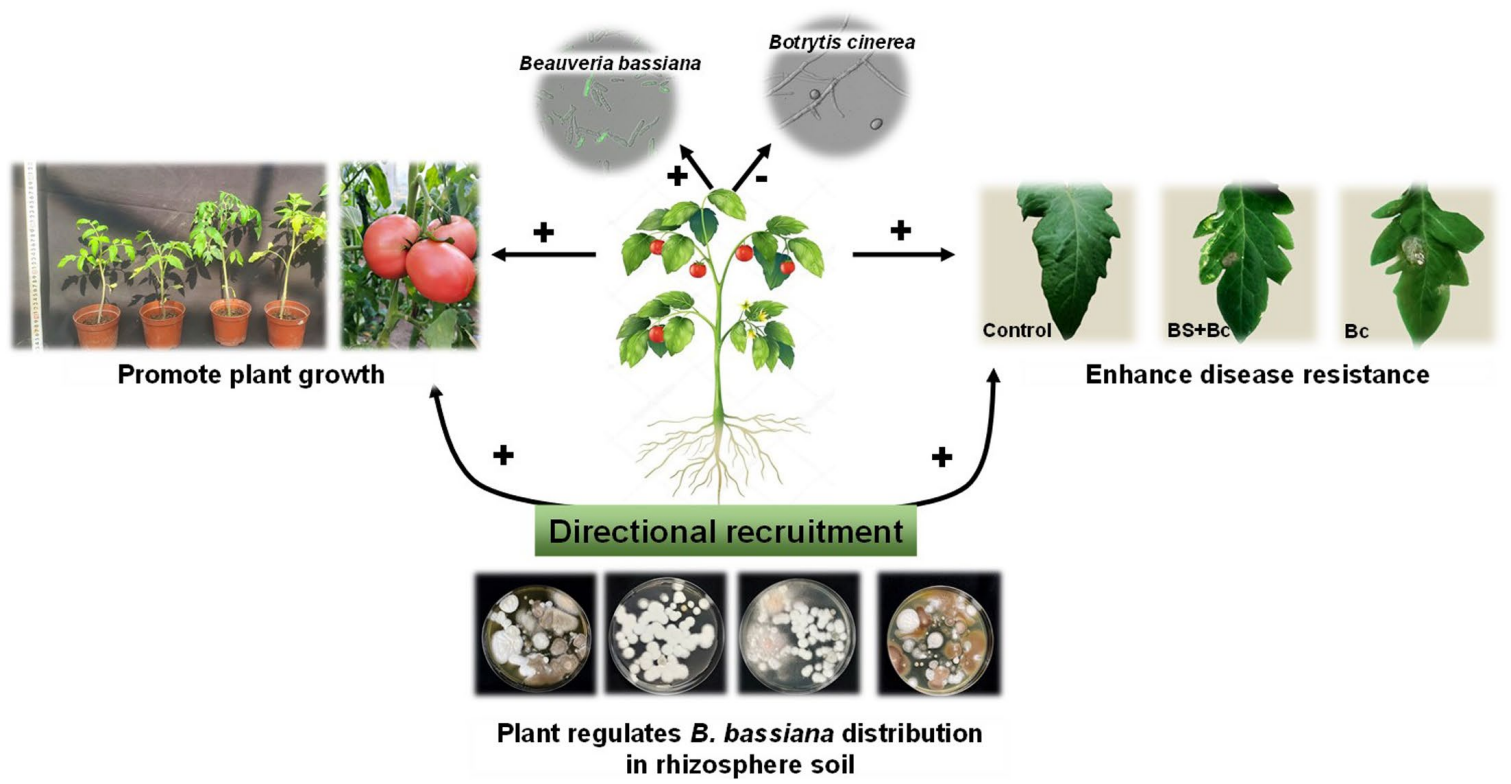
Directional recruitment effects of plant (tomato) on entomopathogenic fungi (*Beauveria bassiana*) under plant pathogen (*Botrytis cinerea*) stress. “+” indicates positive effect, “-” indicates negative effect.

Beneficial plant-associated microbes could stimulate plant growth and enhance resistance to biotic and abiotic stress (Compant et al., 2010; Shikano et al., 2017; Chaudhry et al., 2021). Distinct microbiota interactions in in the leaf compartment influence plant host and shape microbial community structure (Chaudhry et al., 2021). In the present study, plants could “recruit” *B. bassiana* from rhizosphere soil to disease spots as directional effects (Figure 8), and then inhibit pathogens based on the *B. bassiana*-plant interactions, further improving plant growth and resistance to pathogenic microbes.

Numerous studies have shown that EPF are associated directly with plants, and they are considered extremely important partners in plant development. EPF have attracted the interest of researchers in recent years because of the benefits they confer to their hosts, especially in the form of plant growth promotion (Ownley et al., 2010; Barra-Bucarei et al., 2020; Deb et al., 2022). In the present study, *B. bassiana* had positive effects on root length and tomato seedling height after inoculation, which is consistent with the findings of other studies showing that EPF could endophytically colonize and promote plant growth, both in monocots and in dicots (Vega, 2008, 2018; Mantzoukas and Eliopoulos, 2020). For example, EPF has plant growth-enhancing effects (significant increases in plant dry biomass and number of squares) in cotton cultivated under greenhouse conditions (Castillo López and Sword, 2015). In addition, *B. bassiana* boosted spike production in bread wheat, and increased root length and grain yield when inoculated using the “seed dressing” and “soil treatment” methods (Sánchez-Rodríguez et al., 2018). Better plant growth during the early stages may result in an increase in overall plant biomass (Yin et al., 2012; Sui et al., 2020). Similarly, we observed that colonization by endophytic *B. bassiana* could increase tomato yield. Previous studies have proved that the relationship between *B. bassiana* and plants was mutually beneficial symbiotic (Quesada-Moraga, 2020; Sui et al., 2020). *B. bassiana* form symbioses with plants and subsequently transfer nutrient to plants (Behie and Bidochka, 2014; Behie et al., 2017; Krell et al., 2018; Quesada-Moraga, 2020), and plants can “domesticate” *B. bassiana* to enhance its virulence (Sui et al., 2020), but how does the plant balance energy used to constrain the endophyte and plant growth needs to be clarified in the future. In addition, *B. bassiana* usually produces a variety of toxins to parasitize and kill the insects (Wang et al., 2021), however, whether *B. bassiana* metabolize the toxins after entering the plant and their safety have not been reported, which needs to be explored. In summary, EPF may play critical and complex roles as modulators of primary ecological functions to promote plant growth, however, the underlying mechanisms of the positive effects in plants are largely unknown and require further elucidation.

Our results also showed that *B. bassiana* colonization can reduce the incidence of plant disease caused by *B. cinerea* significantly. Ownley et al. (2004), for the first time, reported that *B. bassiana* strain 11–98 colonization could suppress damping-off caused by soil-borne pathogens, *R. solani* and *P. myriotylum*, in tomato, and pre-treatment of cotton seedlings with the same *B. bassiana* strain decreased the severity of bacterial blight caused by *Xanthomonas axonopodis* pv. malvacearum (Xam) (Ownley et al., 2008). There is now substantial evidence that some endophytic fungal entomopathogens may exhibit antagonistic activity against plant pathogens and minimize their adverse effects on host plants (Barra-Bucarei et al., 2020; Canassa et al., 2020). It was found that the activities of two resistance related enzymes in maize, phenylalaninammo-nialyase (PAL) and polyphenoloxidase (PPO), significantly increased following *B. bassiana* inoculation in our previous study (Sui et al., 2020), this indicated that the plant induced resistance system was activated, similar results were seen from Qin et al. (2021) that colonization by *B. bassiana* was shown to trigger both of the salicylic acid (SA) and jasmonate acid (JA) defense pathways benefit for plant resistance to biotic stress. Furthermore, strong evidence suggests that a combination of the mechanisms, rather than a single mechanism, is employed by endophytic fungal entomopathogens against plant pathogens, such as induction of systemic plant resistance, stimulation of plant secondary metabolites, and promotion of plant growth (Vega et al., 2009; Jaber and Ownley, 2017; González-Guzmán et al., 2022).

The present study showed the dynamic distribution of *B. bassi.ana* induced by *B. cinerea*. Specifically, when leaves were infected by pathogens, *B. bassiana* and disease resistance related gene contents in tomato leaves were extremely higher than those in the control treatments. The observation confirmed that plants experiencing biotic or abiotic stress could employ a range of chemical stimuli to recruit beneficial microbes from the environment to enhance their capacity to tolerate stress (Liu et al., 2020). Such a phenomenon in which plants actively seek cooperation with microbes to combat stress is known as the “cry for help” strategy (Bakker et al., 2018). The microbiome has long been recognized as an essential component of the crop ecosystem and is closely linked to plant growth and resistance to disease (Li P. D. et al., 2022). For example, Berendsen et al. (2018) indicate that plants could adjust their root microbiome following pathogen infection and specifically recruit disease resistance-inducing and growth-promoting microbes, which potentially enhance the survival potential of their offspring that would grow in the same soil. Similarly, Chang et al. (2021) showed that *B. bassiana* colonization and *Exserohilum turcicum* infection increased the relative abundance of plant beneficial bacteria (*Burkholderia* and *Pseudomonas*) in maize leaves significantly, with positive biological control and plant growth promotion effects. Our study highlights that plants can regulate EPF distribution in plant tissues and their own defense responses under abiotic stress. However, the inhibitory effects of beneficial microbes against phytopathogens largely depend on their competition for nutrients and niches with the host plant, which could result in insufficient resources for phytopathogens for growth maintenance, in turn inhibiting phytopathogen proliferation and reducing plant disease index and morbidity.

The investigated EPF exhibited multifaceted functions (Vega et al., 2009; Zheng et al., 2023). The present study reveals how *B. bassiana* varies under *B. cinerea* infection and identifies the potential mechanisms *via* which the observed shifts in microbiome could have helped plants cope with pathogen pressure. Our results support the hypothesis that the endophytic *B. bassiana* can be regulated to elicit a directional effect on plant growth and resistance in plant tissue. Overall, our findings illustrate that endophytic EPF have of significance effects on interaction modifications and as ecosystem modulators in plant-microbe-pathogen interactions, these findings indicate an even greater potential of the use of EPF as an ecologically safe strategy of biological control in agroecosystems.

## Data availability statement

The original contributions presented in the study are included in the article/Supplementary material, further inquiries can be directed to the corresponding authors.

## Author contributions

LS, ZZ, and QL conceived and designed the research. YL, LZ, and NL conducted this experiment. LS, LZ, and ZZ analyzed data. LS and ZZ wrote the manuscript. All authors read and approved the manuscript.

## Funding

This work was supported by the Natural Science Foundation of Jilin Province Science and Technology Department (grant number 20220101313JC) and the National Natural Science Foundation of China (grant number 32271683).

## Conflict of interest

The authors declare that the research was conducted in the absence of any commercial or financial relationships that could be construed as a potential conflict of interest.

## Publisher’s note

All claims expressed in this article are solely those of the authors and do not necessarily represent those of their affiliated organizations, or those of the publisher, the editors and the reviewers. Any product that may be evaluated in this article, or claim that may be made by its manufacturer, is not guaranteed or endorsed by the publisher.
