# Supplementary material for: Endophytic Beauveria bassiana promotes plant biomass growth and suppresses pathogen damage by directional recruitment
Source: Front Microbiol. 2023 Aug 16;14:1227269. doi: 10.3389/fmicb.2023.1227269 (PMC10468600; doi:10.3389/fmicb.2023.1227269)
Supplement: Supplementary file 1 [file Table_1.pdf]

Supplementary Material

Endophytic *Beauveria bassiana* promotes plant biomass growth and suppresses pathogen damage by directional recruitment

Li Sui*, Yang Lu*, Qiyun Li, Zhengkun Zhang

First Author*, Co-Author, Co-Author

*** Correspondence:** Corresponding Author: zhanghengkun1980@126.com; qyli1225@126.com

Supplementary Table

Table S1 Gene primer sequences

Gene Name	Primer sequence（5'to 3'）
Actin7 F	GGTATCCACGAGACTACCTACA
Actin7-R	TGCTCATACGGTCAGCAATAC
Bb18SrRNA-F	CAAGTCTGGCAGCAAACGTC
Bb18SrRNA-R	CAGCCACCCTGTGAGATTGT
Bc-F	AACCCGACTTTGGACCTG
Bc-R	TTGCTTCCGATTGATTGC
OXO-F	GGGCTAAATCCACCTCA
OXO-R	GGCACCACGAACATCTC
CHI-F	TGGTATGGCGTAAGTCGGTA
CHI-R	CTTGGAATCAAAGTCCGGTT
atpA-F	AGGCTCATATACGGAACGG
atpA-R	GAGTGAGGCTTATTTGGGTC
